# Genetic links between atopy, allergy, and alopecia areata: insights from a Mendelian randomization study

**DOI:** 10.1186/s13223-024-00892-w

**Published:** 2024-04-27

**Authors:** Wen Xu, Hongyan Zhang, Sheng Wan, Bo Xie, Xiuzu Song

**Affiliations:** 1 School of Medicine, Zhejiang University, Yuhangtang Rd 866, Hangzhou, 310009 People’s Republic of China; 2 Department of Dermatology, Hangzhou Third People’s Hospital, Affiliated Hangzhou Dermatology Hospital, Zhejiang University School of Medicine, Hangzhou Third Hospital Affiliated to Zhejiang Chinese Medical University, West Lake Ave 38, Hangzhou, 310009 People’s Republic of China

**Keywords:** Alopecia areata, Atopy, Allergy, Type II inflammation, Mendelian randomization, GWAS

## Abstract

**Background:**

Alopecia areata (AA), a prevalent form of autoimmune hair loss, has a not well-defined relationship with atopic and allergic disorders, including eczema, hay fever, and asthma.

**Objectives:**

This study aims to elucidate the genetic relationship between atopy, allergies, and alopecia areata (AA) using Mendelian randomization. We hypothesize that atopic and allergic conditions contribute to the genetic predisposition of AA.

**Methods:**

We analyzed extensive genetic data from Genome-wide Association Studies (GWAS) involving over one million individuals. This analysis focused on assessing the genetic correlation between AA and various allergic conditions, including hay fever, eczema, asthma, and allergies to pollen, dust, and cats. The inverse variance weighted method served as our primary analytical tool, complemented by sensitivity analyses to verify the robustness of our results.

**Results:**

Our findings reveal a significant genetic correlation between atopy/allergies and an increased risk of AA. Notably, strong associations were observed for hay fever, eczema, asthma, and specific allergies (pollen, dust, and cats). The sensitivity analyses corroborated these associations, reinforcing the reliability of our primary results.

**Conclusions:**

This study provides compelling genetic evidence of an association between atopic and allergic conditions and the development of AA. These findings suggest that individuals with such conditions may benefit from enhanced surveillance for early signs of AA.

**Supplementary Information:**

The online version contains supplementary material available at 10.1186/s13223-024-00892-w.

## Introduction

Alopecia areata (AA), an autoimmune disorder resulting in non-scarring hair loss, impacts about 2% of the global population [1], predominantly affecting individuals under 40 years, with a noticeable prevalence in Asian communities [2]. The condition’s impact on the quality of life is substantial, akin to that of severe dermatological disorders, influencing both physical appearance and psychological health [3]. AA is often accompanied by other autoimmune diseases and a higher predisposition to mental health issues, such as depression, anxiety, and sleep disorders [4]. Recognizing these broad impacts highlights the importance of identifying risk factors and developing targeted treatment approaches to mitigate AA's effects.

Historically, AA has been primarily associated with Th1/IFN-γ overactivation, emphasizing type I over type II inflammatory responses (atopy and allergies) [5]. However, recent studies have increasingly explored the association between AA and allergic conditions, like eczema, hay fever, and asthma [6]. While some studies have found a positive correlation between these conditions and AA, the evidence remains inconclusive, with large-scale studies suggesting a complex interplay between the number of atopic conditions experienced and the risk of developing AA [7]. Additionally, the relationship with common allergens such as dust mites, cats, and pollen warrants further investigation [8]. Currently, there's no definitive evidence establishing a causal link between atopy, allergies, and AA.

Traditional research methods face challenges in fully excluding confounding factors, potentially leading to biased conclusions [9]. The implementation of randomized controlled trials in this context is both logistically challenging and ethically complex. In response, Mendelian randomization (MR) is increasingly being utilized to derive causal inferences between risk factors and diseases [10]. MR uses genetic variants as proxies for environmental exposures, allowing for the evaluation of exposure-disease relationships while minimizing confounding. It provides a framework for assessing the potential causal relationship between atopy, allergies, and AA [11].

This study utilizes MR to investigate the causal impact of atopy and allergies on AA, leveraging data from genome-wide association studies (GWAS) [12]. Our objective is to explore the role of type II inflammatory responses in AA, identify susceptible populations, and inform personalized treatment strategies for AA.

## Materials and methods

### MR design

Our study adopted a two-sample MR approach to investigate the causal effects of atopic and allergic conditions—including hay fever, eczema, asthma, and allergies to cats, dust, and pollen—on AA incidence. We used single nucleotide polymorphisms (SNPs) from GWAS summary data as instrumental variables. These SNPs, reflecting the random allocation of genetics, help address confounders like age and sex [13]. Our MR analysis was grounded on three assumptions: the genetic instruments strongly predict the exposure, are free from confounders, and influence the outcome solely through the exposure [14].

### Exposure GWAS data

We sourced atopy and allergy GWAS data from the Social Science Genetic Association Consortium (SSGAC) [15], including data from the UK Biobank and 23andMe but excluding FinnGen. We opted for multi-trait GWAS given its superior predictive power over its single-trait counterpart [16]. We focused on six phenotypes: hay fever, eczema, asthma, and allergies to cats, dust mites, and pollen. The effective sample sizes ranged from 254,386 to 1,214,626, with all participants being of European descent. Diagnosis criteria were based on ICD-10 or self-reported surveys [15] (Table 1).

**Table 1:** Data source of the atopy and AA

Trait	Consortium	Sample size	Ancestry	PMID	Web source
Hay fever	SSGAC	518,480	European	34140656	www.thessgac.org/data
Eczema	SSGAC	1,214,626	European	34140656	www.thessgac.org/data
Asthma	SSGAC	559,863	European	34140656	www.thessgac.org/data
Pollen allergy	SSGAC	366,535	European	34140656	www.thessgac.org/data
Dust allergy	SSGAC	356,879	European	34140656	www.thessgac.org/data
Cat allergy	SSGAC	254,386	European	34140656	www.thessgac.org/data
AA	FinnGen	361,822	European	36653562	www.finngen.fi/en

“Dust allergy” means allergy to dust mites

*AA* alopecia areata

### Outcome GWAS data

AA data came from the latest FinnGen database release (DF9—as of May 11, 2023) [17], encompassing 682 AA patients and 361,140 controls of European ancestry (Table 1). Diagnoses adhered to ICD revisions 8 through 10. We noted an average patient age of 42.12 years, with a slight age variance between genders. To mitigate population heterogeneity bias, we only used SNPs from European ancestry GWAS data.

### Instrumental variable selection

Our SNP selection involved multiple rigorous steps. Starting with genome-wide significant SNPs (*p* < 5 × 10^–8^), we excluded those in linkage disequilibrium (R^2^ < 0.001, window size = 10,000 kb) [18] or additionally, weak SNPs (F-statistic < 10) [19]. Post-harmonization, we further eliminated palindromic and ambiguous SNPs (those with a minor allele frequency (MAF) > 0.01) [20]. The MR-Pleiotropy Residual Sum and Outlier (MR-PRESSO) analysis [21], helped re-evaluate and exclude SNPs potentially influenced by pleiotropy, ensuring a robust set of instrumental variables for each atopic phenotype.

### MR analysis

Addressing pleiotropy concerns, we utilized five MR methods: inverse variance weighted (IVW), MR-Egger regression, weighted median, maximum likelihood ratio, and weighted mode. And the IVW method, our primary analysis technique. Each method offers unique strengths and mitigates specific biases, ensuring a comprehensive examination of the causal effects [22, 23].

### Sensitivity analysis

To ensure the validity of our primary results, we performed sensitivity analyses including heterogeneity tests (Cochran’s Q tests and MR-PRESSO global tests) and horizontal pleiotropy tests (MR-Egger intercept tests and leave-one-out analyses). These analyses helped identify sensitivity and the influence of individual SNPs on the causal estimate, enhancing the reliability of our findings [24].

### Statistical analysis

Statistical significance was set at *p* < 0.05. We expressed MR estimates as odds ratios (ORs) with 95% confidence intervals (CIs), indicating the relative risk of AA associated with each atopic phenotype. Analyses were conducted using ‘Two Sample MR’ (version 0.5.7) and ‘MRPRESSO’ (version 1.0) packages in R (version 4.3.1), ensuring rigorous assessment of the MR estimates and identification of pleiotropic outliers [25].

## Results

### MR estimates

**Fig. 1:**
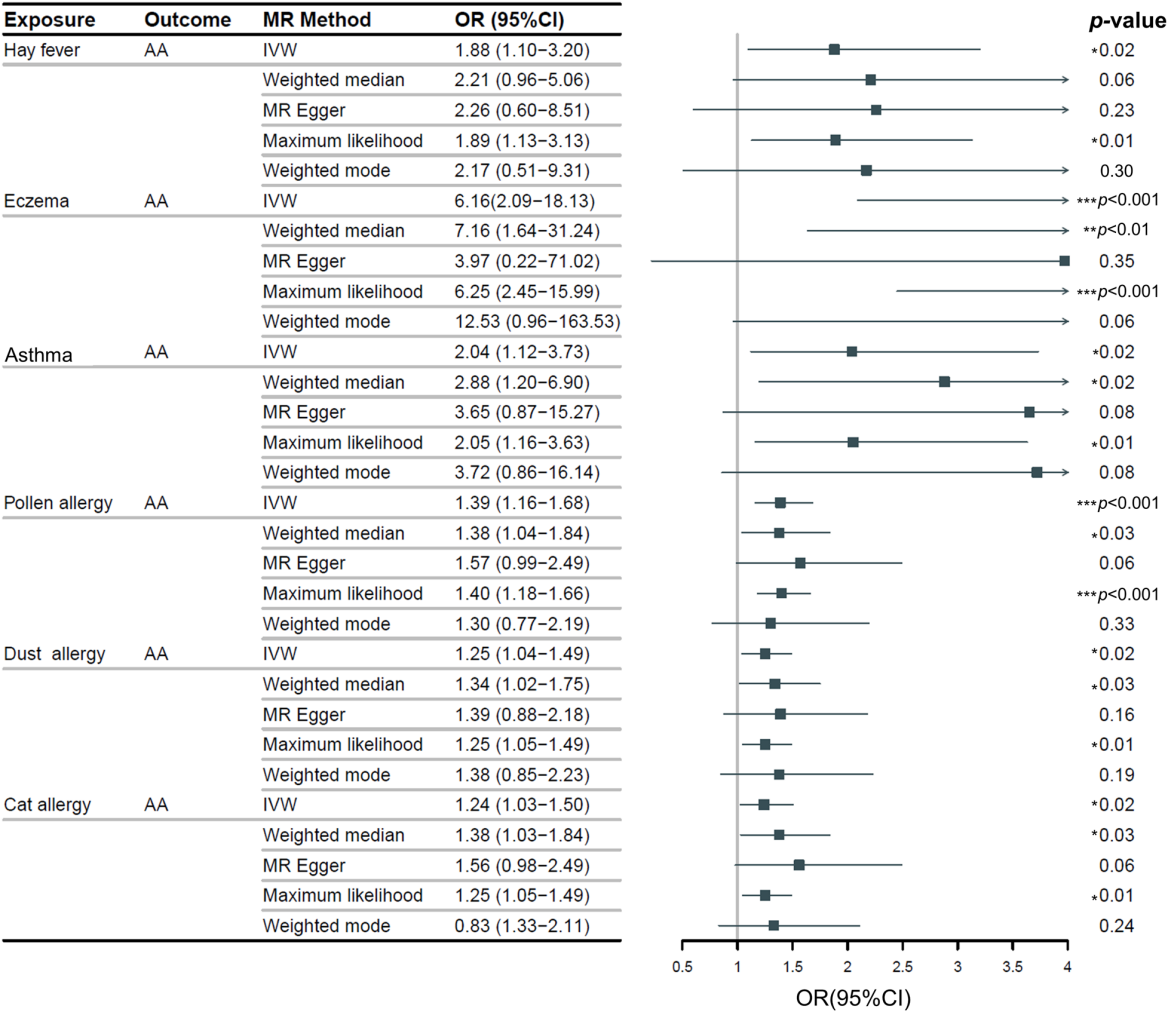
MR estimates for the association between atopy, allergies and AA. “ → ” indicates exceeding the marked range. “Dust allergy” means allergy to dust mites. “*” means *p*-value is less than 0.05. “**” means *p*-value is less than 0.01. “***” means *p*-value is less than 0.001. *MR* Mendelian randomization, *OR* odds ratio, *CI* confidence interval, *AA* alopecia areata, *IVW* inverse variance weighted

**Fig. 2:**
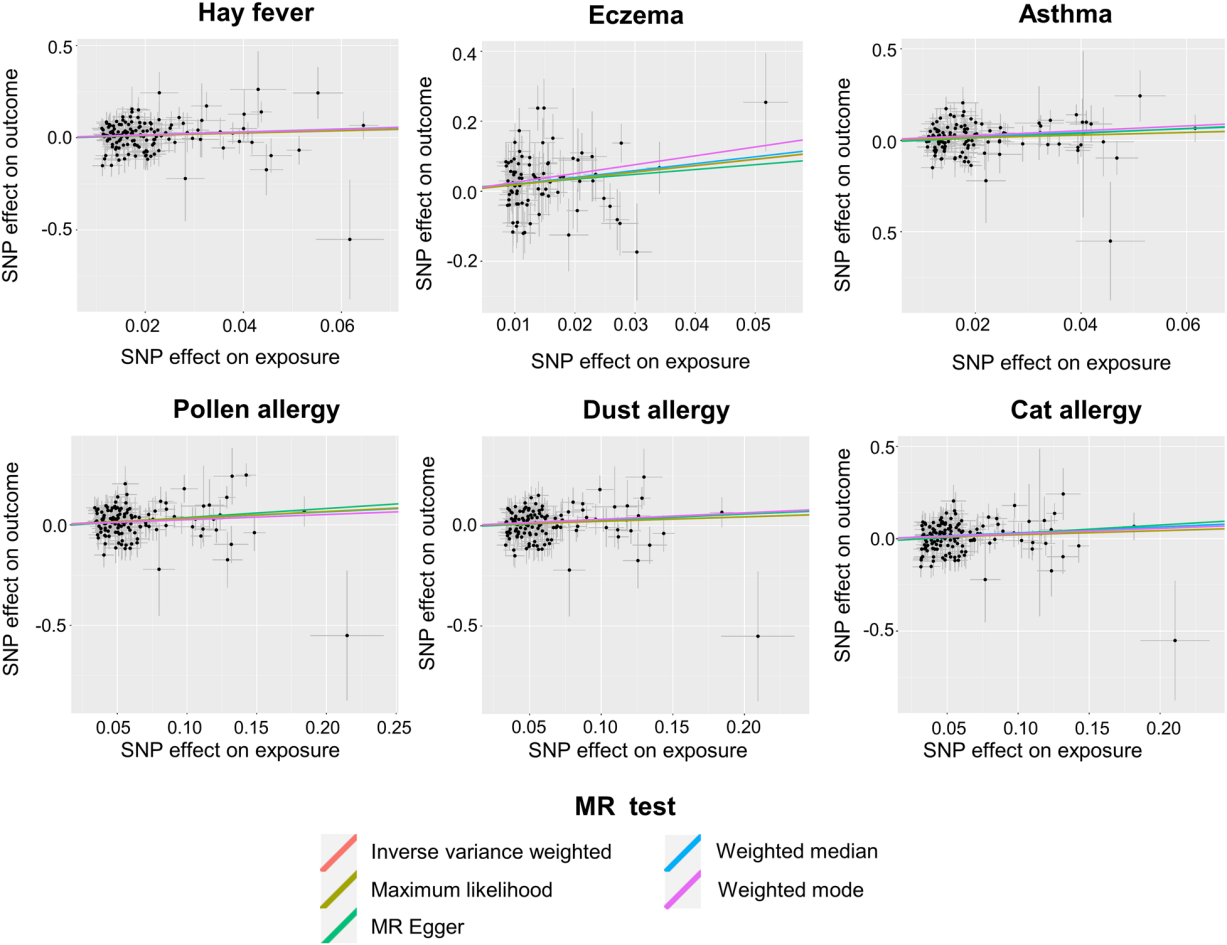
Scatter plot analyses illustrating the causative relationship between the six atopic and allergic conditions and AA. “Dust allergy” means allergy to dust mites. *MR* Mendelian randomization, *AA* alopecia areata

The IVW method, our primary analysis technique, indicated a significant increase in AA risk associated with hay fever (OR = 1.88, 95% CI 1.10–3.20; *p* = 0.02), eczema (OR = 6.16, 95% CI 2.09–18.13; *p* < 0.001), asthma (OR = 2.04, 95% CI 1.12–3.73; *p* = 0.02), pollen allergy (OR = 1.39, 95% CI 1.16–1.68; *p* < 0.001), dust allergy (OR = 1.25, 95% CI 1.04–1.49; *p* = 0.02), and cat allergy (OR = 1.24, 95% CI 1.03–1.50; *p* = 0.02) (Fig. 1). The corresponding scatter plot was shown in Fig. 2, and all analysis methods showed consistent directionality. These associations were corroborated by the maximum likelihood (ML) model, emphasizing the notable link between these conditions and AA. While the weighted median approach found no significant link for hay fever, it did confirm the connection with the other conditions, aligning with the general trend observed in the IVW model. The MR-Egger and weighted mode methods further identified these six conditions as risk factors for AA, although their statistical significance was not as pronounced.

### Sensitivity analyses

The MR-Egger intercept did not indicate the presence of horizontal pleiotropy (*p* > 0.05 for all exposures), suggesting that our results are free from bias related to pleiotropy. Despite the detection of heterogeneity in eczema exposure (*p* < 0.05), we applied a random effects IVW model to mitigate this issue (Table 2). Despite the detection of heterogeneity in eczema exposure, the random effects IVW was utilized to perform the MR analysis and balance this heterogeneity. The absence of horizontal pleiotropy bias in this context was reaffirmed by leave-one-out analysis, and forest plots (Additional file 1: Figs. S1–S6) confirmed that no single SNP disproportionately influenced our results, further attesting to the reliability of our findings.

**Table 2:** Sensitivity analysis of the causal association between atopy and the risk of AA

Exposure	Outcome	Cochran Q test		MR-Egger		MR-PRESSO (global test)	
		Q value	*p* value	Intercept	*p* value	RSSobs	*p* value
Hay fever	AA	155.07	0.17	− 4.22E−03	0.77	157.34	0.16
Eczema	AA	112.20	0.02	6.88E−03	0.75	114.84	0.02
Asthma	AA	133.78	0.15	− 0.01	0.38	136.16	0.16
Pollen allergy	AA	145.93	0.11	− 8.33E−03	0.58	149.20	0.10
Dust allergy	AA	132.83	0.37	− 7.27E−03	0.61	135.05	0.37
Cat allergy	AA	166.04	0.08	− 0.01	0.30	168.37	0.10

“Dust allergy” means allergy to dust mites

*AA* alopecia areata, *MR* Mendelian randomization, *MR-PRESSO* MR-Pleiotropy Residual Sum and Outlier (MR-PRESSO)

## Discussion

This investigation harnessed extensive GWAS data and multiple MR methodologies to explore the potential causality between atopy, allergy, and AA susceptibility. Our data elucidates a significant causal association with conditions like asthma, hay fever, eczema, and allergies to pollen, dust, and cats, each contributing to an elevated AA risk. This emphasizes the importance of integrating a patient’s atopic and allergic history into AA management, highlighting the significant role of Type II inflammatory pathways.

AA's etiology is believed to involve immune events, leading to hair follicle autoantigen exposure [26]. While Th1 and Th2 pathways are implicated in AA's pathogenesis [5], observational studies, limited by their inability to confirm causality, have indicated a correlation between atopy, allergy, and AA. MR offers a more refined approach to determine causality, potentially directing treatment strategies.

Notably, clinical evidence points towards the efficacy of dupilumab, a Type II inflammatory response inhibitor, in treating AA, especially in patients with concurrent allergic conditions [27]. However, instances where dupilumab might have induced AA during atopic dermatitis treatment have been reported, though AA typically resolved with continued treatment [28]. This suggests that atopic and allergic profiles should be considered in AA management, supporting the notion that targeting Th2 pathways could benefit AA treatment, particularly in patients with a history of allergies.

Nevertheless, the study’s scope, limited to participants of European descent and a selection of common diseases and allergens, may restrict its generalizability. Additionally, the nature of MR, while offering insight into causality, does encounter challenges in fully differentiating mediation effects from pleiotropy.

In conclusion, our findings substantiate the causal link between allergies, atopy, and AA, using large-scale genetic data. Further investigations into the specific mechanisms of the Th2 inflammatory pathway in AA and its interaction with the Th1 pathway are warranted. Considering the robust connection between AA and allergies, it's imperative to factor in patients' atopic and allergic histories when devising personalized treatment plans.

### Supplementary Information

**Additional file 1: Figure S1.** Leave-one-out analysis (**a**) and forest plot (**b**) for hay fever on AA risk. *AA* alopecia areata, *MR* Mendelian randomization. **Figure S2.** Leave-one-out analysis (**a**) and forest plot (**b**) for eczema on AA risk. *AA* alopecia areata, *MR* Mendelian randomization. **Figure S3.** Leave-one-out analysis (**a**) and forest plot (**b**) for asthma on AA risk. *AA* alopecia areata, *MR* Mendelian randomization. **Figure S4.** Leave-one-out analysis (**a**) and forest plot (**b**) for pollen allergy on AA risk. *AA* alopecia areata, *MR* Mendelian randomization. **Figure S5.** Leave-one-out analysis (**a**) and forest plot (**b**) for dust mite allergy on AA risk. *AA* alopecia areata, *MR* Mendelian randomization. **Figure S6.** Leave-one-out analysis (**a**) and forest plot (**b**) for cat allergy on AA risk. *AA* alopecia areata, *MR* Mendelian randomization.

## Data Availability

Data pertaining to atopy and allergies for the present study was procured from the Social Science Genetic Association Consortium (SSGAC), accessible via their official webpage, https://www.thessgac.com/papers/. Data concerning alopecia areata (AA) can be sourced from the FinnGen Consortium, available at their official website, https://www.finngen.fi/en.

## Abbreviations

AA: Alopecia areata
GWAS: Genome-wide Association Studies
MR: Mendelian randomization
IVW: Inverse variance weighted
SNPs: Single nucleotide polymorphisms
SSGAC: Social Science Genetic Association Consortium
ICD-8, ICD-9, and ICD-10: International Classification of Diseases, encompassing the 8th, 9th, and 10th revisions
MAF: Minor allele frequency
MR-PRESSO: MR-Pleiotropy Residual Sum and Outlier
OR: Odds ratio
CI: Confidence interval
