# Supplementary material for: Genetic links between atopy, allergy, and alopecia areata: insights from a Mendelian randomization study
Source: Allergy Asthma Clin Immunol. 2024 Apr 27;20:32. doi: 10.1186/s13223-024-00892-w (PMC11055271; doi:10.1186/s13223-024-00892-w)
Supplement: Supplementary file 1 — Additional file 1: Figure S1. Leave-one-out analysis (a) and forest plot (b) for hay fever on AA risk. AA alopecia areata, MR Mendelian randomization. Figure S2. Leave-one-out analysis (a) and forest plot (b) for eczema on AA risk. AA alopecia areata, MR Mendelian randomization. Figure S3. Leave-one-out analysis (a) and forest plot (b) for asthma on AA risk. AA alopecia areata, MR Mendelian randomization. Figure S4. Leave-one-out analysis (a) and forest plot (b) for pollen allergy on AA risk. AA alopecia areata, MR Mendelian randomization. Figure S5. Leave-one-out analysis (a) and forest plot (b) for dust mite allergy on AA risk. AA alopecia areata, MR Mendelian randomization. Figure S6. Leave-one-out analysis (a) and forest plot (b) for cat allergy on AA risk. AA alopecia areata, MR Mendelian randomization. [file 13223_2024_892_MOESM1_ESM.docx]

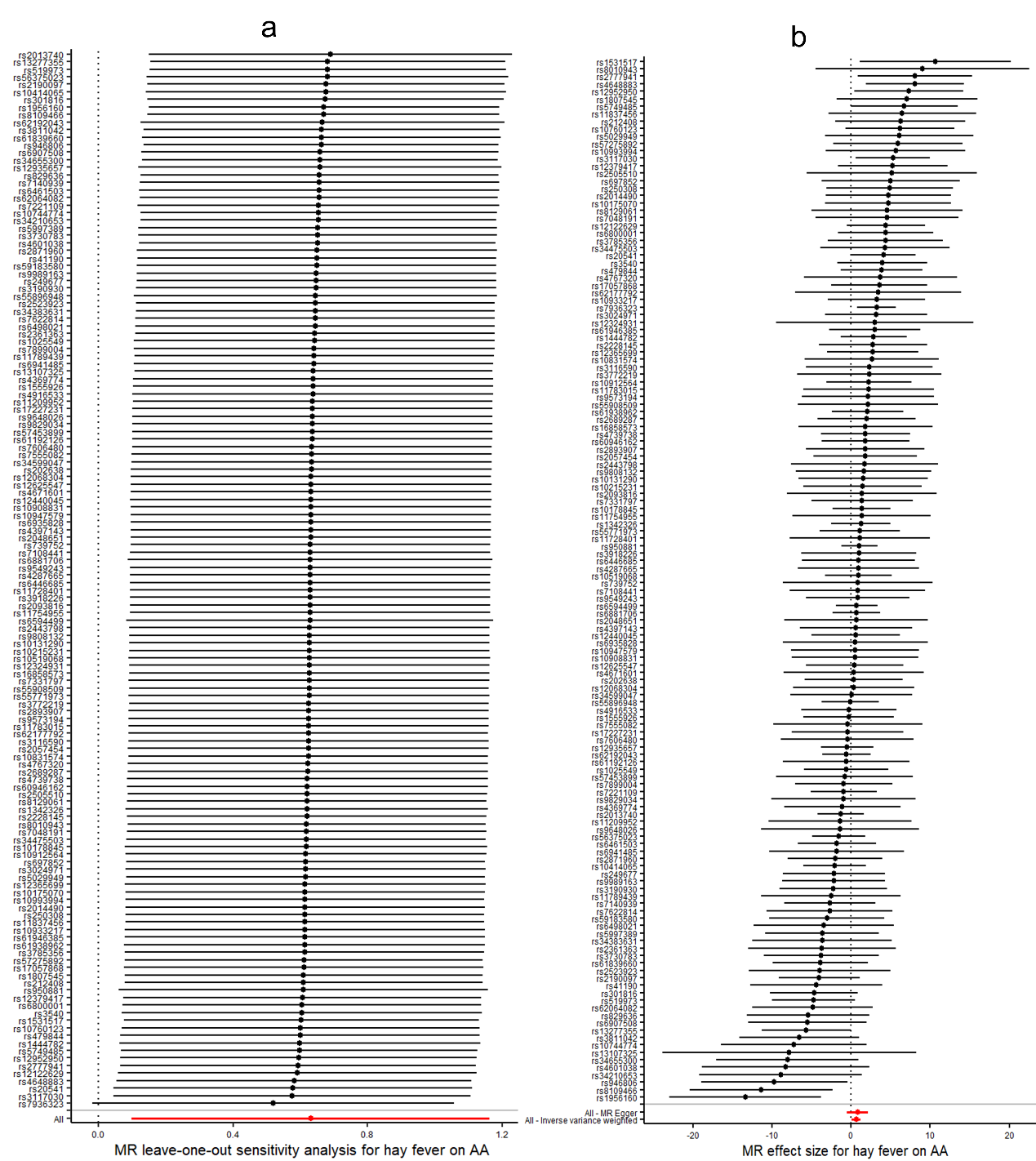


Figure S1. leave-one-out analysis(a) and forest plot(b) for hay fever on AA risk.

AA, alopecia areata. MR, Mendelian randomization.
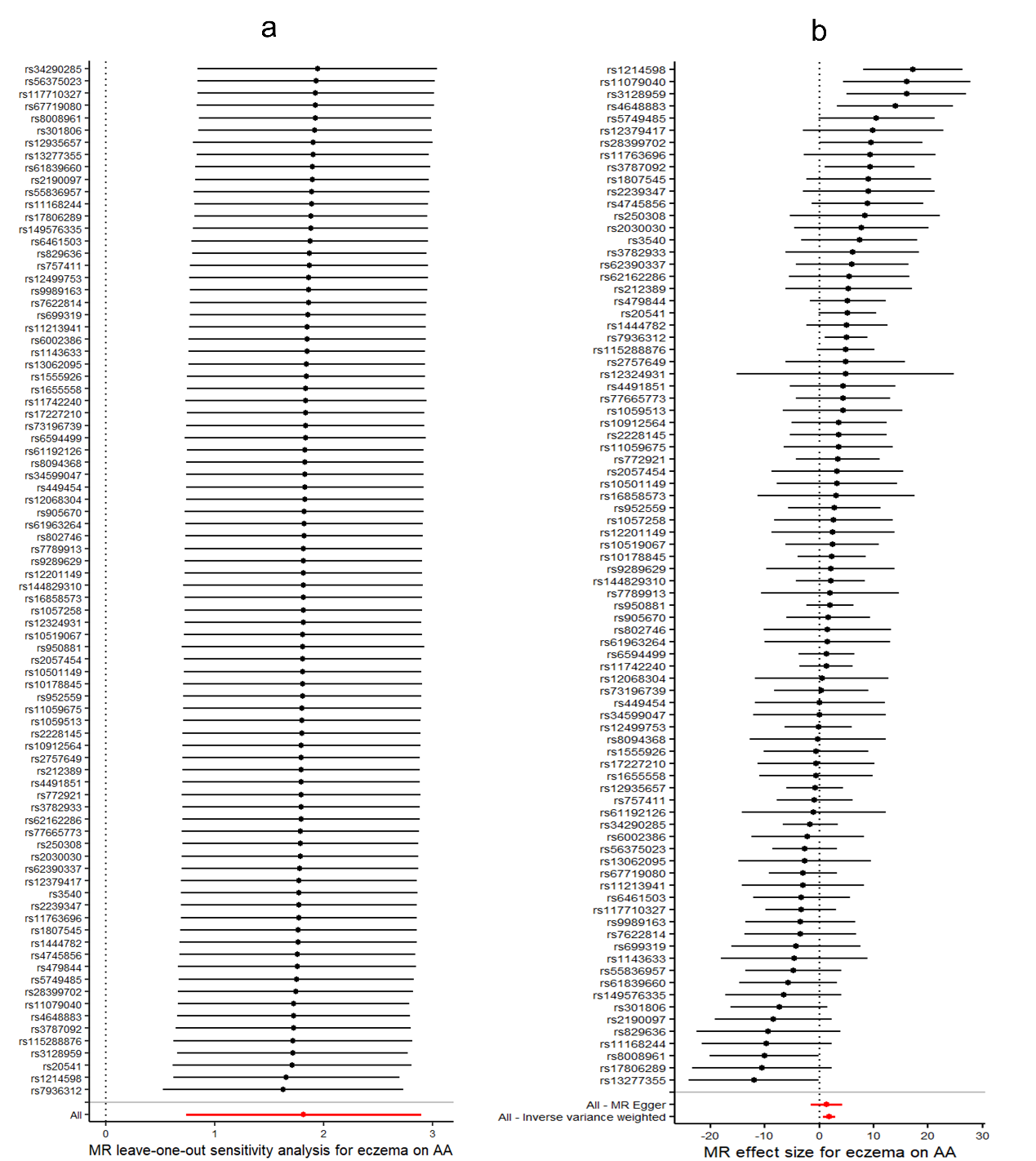


Figure S2. leave-one-out analysis(a) and forest plot(b) for eczema on AA risk.

AA, alopecia areata. MR, Mendelian randomization.


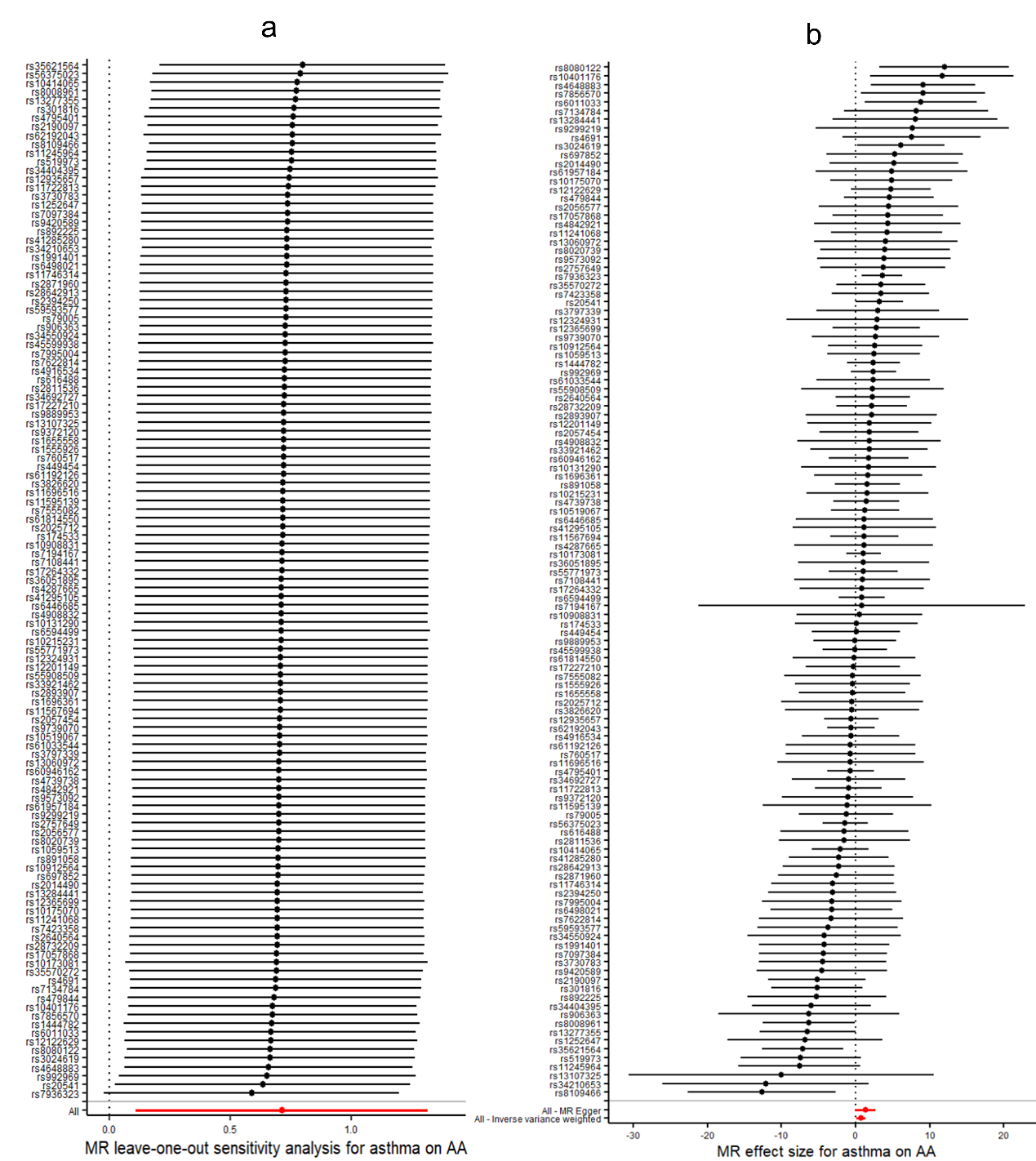


Figure S3. leave-one-out analysis(a) and forest plot(b) for asthma on AA risk.

AA, alopecia areata. MR, Mendelian randomization.


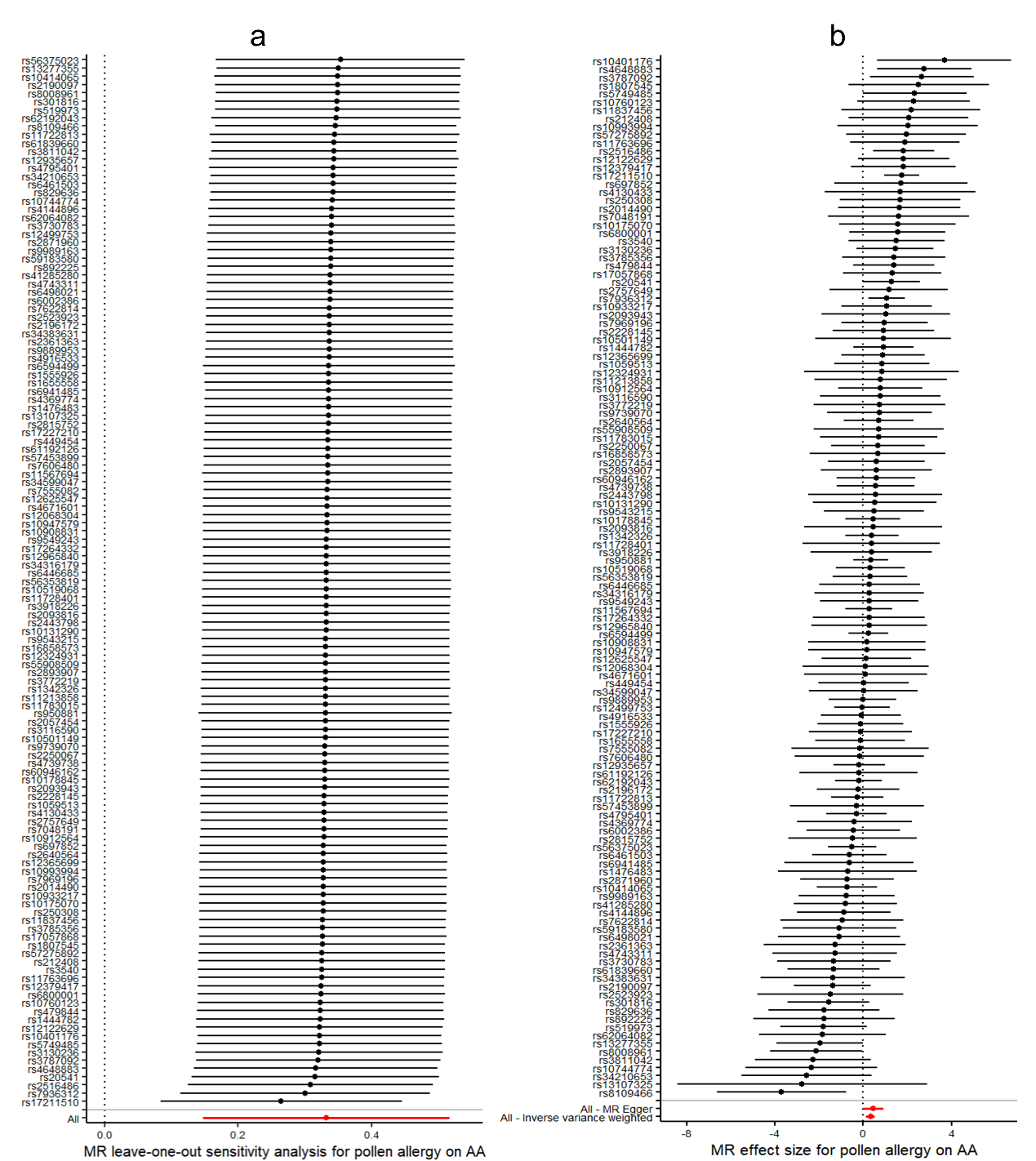


Figure S4. leave-one-out analysis(a) and forest plot(b) for pollen allergy on AA risk.

AA, alopecia areata. MR, Mendelian randomization.


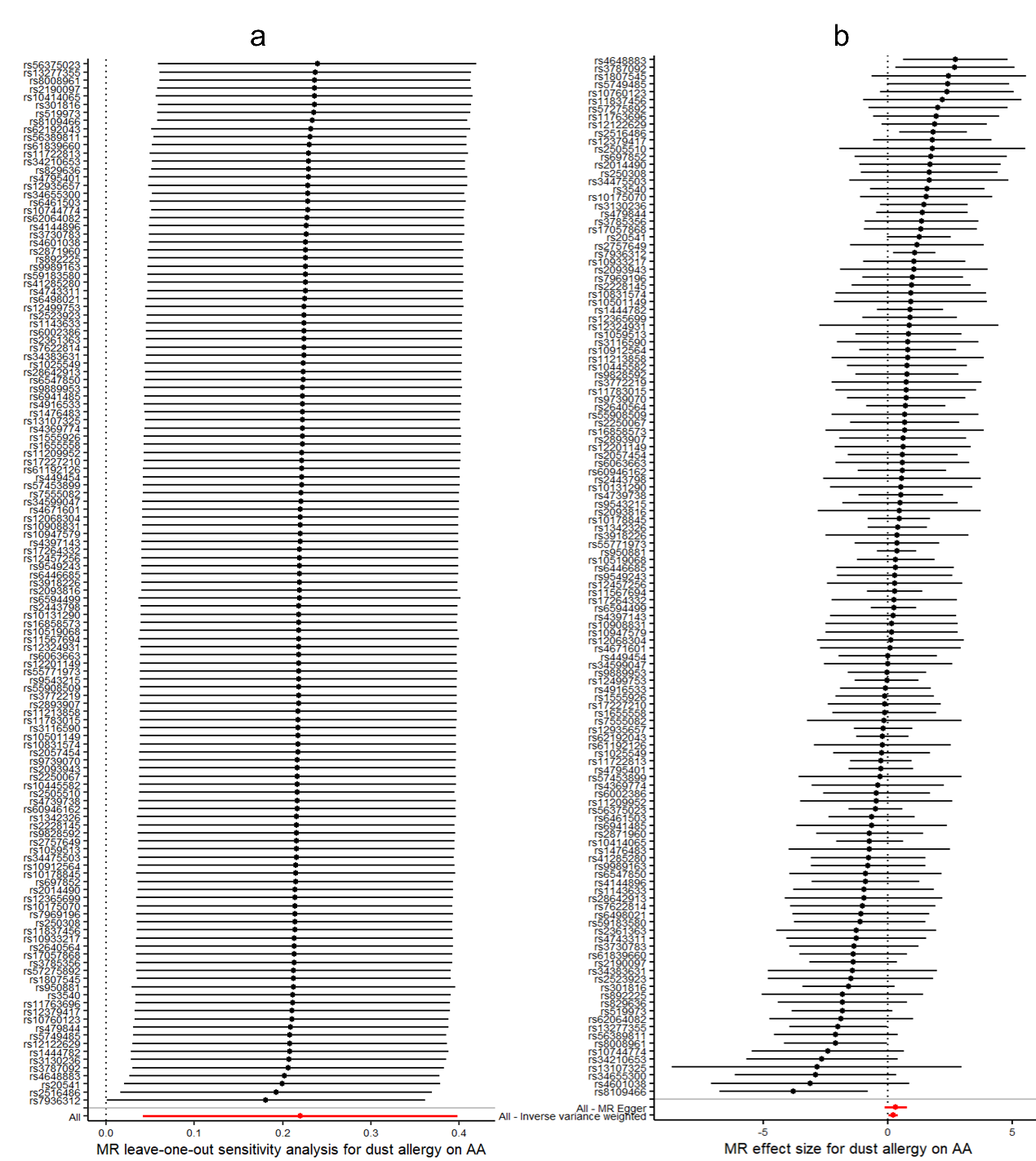


Figure S5. leave-one-out analysis(a) and forest plot(b) for dust mite allergy on AA risk.

AA, alopecia areata. MR, Mendelian randomization.


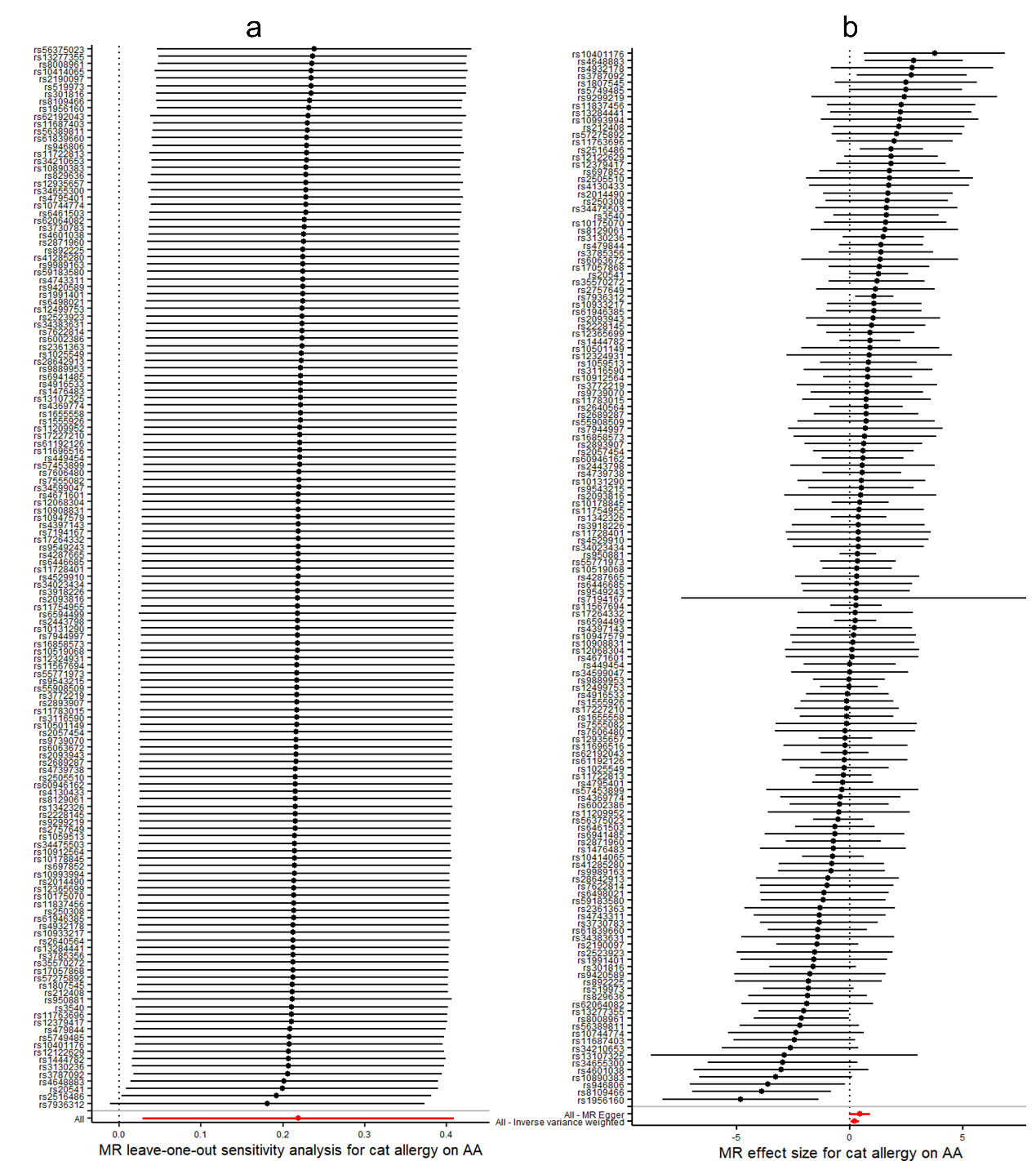


Figure S6. leave-one-out analysis(a) and forest plot(b) for cat allergy on AA risk.

AA, alopecia areata. MR, Mendelian randomization.
